# EB1 and cytoplasmic dynein mediate protrusion dynamics for efficient 3-dimensional cell migration

**DOI:** 10.1096/fj.201700444RR

**Published:** 2017-11-02

**Authors:** Hasini Jayatilaka, Anjil Giri, Michelle Karl, Ivie Aifuwa, Nicholaus J. Trenton, Jude M. Phillip, Shyam Khatau, Denis Wirtz

**Affiliations:** * Department of Chemical and Biomolecular Engineering, The Johns Hopkins University, Baltimore, Maryland, USA;; † Johns Hopkins Physical Sciences–Oncology Center, The Johns Hopkins University, Baltimore, Maryland, USA;; ‡ Department of Bioengineering, Rice University, Houston, Texas, USA;; § Division of Hematology and Oncology, Department of Medicine, Weill Cornell Medicine, New York, New York, USA;; ¶ Department of Pathology, The Johns Hopkins School of Medicine, Baltimore, Maryland, USA;; ‖ Department of Oncology, The Johns Hopkins School of Medicine, Baltimore, Maryland, USA;; # Sydney Kimmel Comprehensive Cancer Center, The Johns Hopkins School of Medicine, Baltimore, Maryland, USA

**Keywords:** RhoA, paclitaxel, microtubule

## Abstract

Microtubules have long been implicated to play an integral role in metastatic disease, for which a critical step is the local invasion of tumor cells into the 3-dimensional (3D) collagen-rich stromal matrix. Here we show that cell migration of human cancer cells uses the dynamic formation of highly branched protrusions that are composed of a microtubule core surrounded by cortical actin, a cytoskeletal organization that is absent in cells on 2-dimensional (2D) substrates. Microtubule plus-end tracking protein End-binding 1 and motor protein dynein subunits light intermediate chain 2 and heavy chain 1, which do not regulate 2D migration, critically modulate 3D migration by affecting RhoA and thus regulate protrusion branching through differential assembly dynamics of microtubules. An important consequence of this observation is that the commonly used cancer drug paclitaxel is 100-fold more effective at blocking migration in a 3D matrix than on a 2D matrix. This work reveals the central role that microtubule dynamics plays in powering cell migration in a more pathologically relevant setting and suggests further testing of therapeutics targeting microtubules to mitigate migration.—Jayatilaka, H., Giri, A., Karl, M., Aifuwa, I., Trenton, N. J., Phillip, J. M., Khatau, S., Wirtz, D. EB1 and cytoplasmic dynein mediate protrusion dynamics for efficient 3-dimensional cell migration.

Cell migration is critical in a myriad of physiologic and pathologic phenomena, including embryonic development, immunologic responses, and cancer metastasis (1–3). Rapid assembly and reorganization of the cytoskeletal proteins F-actin and microtubule are the driving factors that generate the necessary forces for cell migration and maintenance of cell polarity, at least for migration on 2-dimensional (2D) substrates (1, 4, 5). Much of what we know about human cell migration has stemmed from studies on 2D flat substrates, which do not recapitulate the *in vivo* conditions that migrating cells are exposed to (6, 7). *In vivo*, post epithelial-to-mesenchymal transition fibrosarcoma cells (used extensively in cell migration studies) rapidly encounter a 3-dimensional (3D) environment comprised of extracellular matrix molecules, particularly type I collagen (8, 9).

Recent studies have shown that the key proteins and organelles that play a critical role in conventional 2D migration do not necessarily play a significant role in 3D migration and *vice versa*. For instance, the Arp2/3 complex–based module for dendritic assembly of F-actin and the associated regulatory/activator proteins such as N-WASP and Cdc42 have remarkably different effects on cell migration in 2- and 3D environments (5, 10). Similarly, focal adhesion proteins, such as vinculin and p130Cas, play critical roles in 3D migration that are not necessarily predicted by the 2D case (5, 11, 12).

The role of microtubules and associated regulatory and motor proteins in 3D migration is largely unknown. Findings from 2D cell migration studies suggest that microtubules are dynamic polymers that cycle between phases of growth and catastrophe. This dynamic structure is maintained by the coordinated activity of a large number of proteins, prominently including end-binding 1 (EB1) and dynein [light intermediate chain 2 (LIC2) and heavy chain 1 (HC1)]. EB1 is a highly conserved microtubule tip-tracking protein that directly binds to a structural motif on the growing end (plus-end) of microtubules and is known to stabilize and stimulate growth at the plus-end (13–18). It also possesses a domain that controls the binding and activity of other microtubule-binding proteins and thus is speculated to be a master regulator of microtubule dynamics (15, 19). Cytoplasmic dynein is a minus-end directed motor protein that mediates diverse cellular processes, including the generation of forces important for cell migration (20, 21). The role of EB1 and dynein on the regulation of cell migration in 3D collagen I matrices is currently unknown.

In this study we demonstrate that cell migration of human fibrosarcoma cells embedded in a type I 3D collagen matrix is regulated by microtubule plus-end tracking protein EB1 and by the motor protein dynein subunits LIC2 and HC1, which do not regulate 2D migration. This regulation is modulated by protrusion branching through differential assembly dynamics of microtubules *via* RhoA (22). Our findings suggest that tumor cells exploit the dynamic formation of highly branched protrusions that are composed of a microtubule core surrounded by cortical actin. This cytoskeletal organization is absent in cells placed on 2D substrates. Furthermore, we observe different migratory phenotypes when cancer cells in 2- and 3D are treated with the microtubule-depolymerizing drug nocodazole and the microtubule-stabilizing drug taxol. Both cancer agents are more effective on matrix-embedded cells than cells on 2D substrates. The results of this study demonstrate that microtubule dynamics may play a significant role in driving cancer cell migration than 2D assays have revealed in this more pathologically relevant setting. These results further suggest testing of therapeutics targeting microtubules to mitigate migration.

## MATERIALS AND METHODS

### Cell culture

Human fibrosarcoma cells (HT1080) (American Type Culture Collection, Manassas, VA, USA) were grown in DMEM (Mediatech, Herndon, VA, USA) supplemented with 10% fetal bovine serum (FBS) (Hyclone Laboratories, Logan, UT, USA) and 50 μg/ml gentamicin (Quality Biologic, Gaithersburg, MD, USA) as antibiotic. WI-38 cells were grown in Minimum Essential Medium (Mediatech) containing 10% FBS (Hyclone Laboratories) and 100 U penicillin/100 µg streptomycin (MilliporeSigma, St. Louis, MO, USA) per milliliter of medium. For protein depletion, HT1080 cells were selected and maintained in medium containing 3 µg/ml puromycin. For culture and live-cell imaging, all cells were maintained in a humidified incubator at 37°C and 5% CO_2_.

### Depletion of EB1, LIC2, and HC1 proteins

Short hairpin RNA (shRNA) constructs against target genes were cotransfected with the packaging plasmids pMD.G VSV-G and pCMVΔR8.91 using Lipofectamine 2000 (Thermo Fisher Scientific, Waltham, MA, USA). Briefly, 293T cells were grown to ∼90% confluency, and a mixture of pMD.G VSV-G, pCMVΔR8.91, and shRNA construct at a 1:8:6 ratio was added to the cells. 293T cells were then incubated with the mixture for 6 h, and the transfection mixture containing medium was replaced with fresh medium.

The lentivirus-containing medium was harvested twice at 24 and 48 h after transfection and filtered through a 0.4-µm filter to remove cell debris. Two milliliters of virus containing filtrate mixed with 1 ml of fresh medium and the polycationic peptide protamine sulfate (10 µg/ml final concentration) was added to ∼60% confluent HT1080 cells and incubated for 8 h. The medium containing the viral vectors was replaced with medium containing 3 µg/ml puromycin for selection. The medium was replaced every 3–4 d thereafter.

Five different shRNAs for each gene were tested, and shRNAs showing at least 85% knockdown were used for subsequent studies. All the shRNAs used in this study were obtained from MilliporeSigma. The shRNAs used for this study include:

*EB1 sh62140*: GCAGCAGGTCAACGTATTGAAC
*EB1 sh62142*: GTTCAGTGGTTCAAGAAGTTTC
*LIC2 sh116993*: CCTCGACTTGTTGTATAAGTAC
*LIC2 sh116996*: GAAAGCCAGACTCTATGGTAAC
*HC1 sh116323*: CCCGTGATTGATGCAGATAAAC
*HC1 sh116324*: GCAGCCAATGACAAGCTGAAAC

We used a scrambled shRNA sequence (CCTAAGGTTAAGTCGCCCTCGC) (plasmid 1864; Addgene, Cambridge, MA, USA) as a control.

The level of protein depletion was confirmed by Western blotting. The blots were incubated at 4°C with the following antibodies: mouse anti-human EB1 (1:1000; Cell Signaling Technology, Danvers, MA, USA), rabbit anti-human LIC2 (1:1000; kindly provided by Richard Vallee, Columbia University, New York, NY, USA), and rabbit anti-human HC1 (1:500; Proteintech, Chicago, IL, USA). β-Actin levels (1:2500; Santa Cruz Biotechnology, Dallas, TX, USA) in the cell were used as controls.

Quantitative RT-PCR was performed on these cell lines as a supplement to the Western blot to verify that the knockdowns were successful. Total RNA isolation was achieved with Trizol (Thermo Fisher Scientific) and RNA MiniPrep Kit (Zymo Research, Irvine, CA, USA). cDNA was synthesized using the iScript cDNA Synthesis Kit (Bio-Rad, Hercules, CA, USA). Quantitative RT-PCR was then performed using this cDNA, accompanied by the appropriate oligonucleotide primers listed below (with GAPDH used as the reference gene) and iTaq Universal SYBR Green Supermix (Bio-Rad). The data were analyzed using Bio-Rad CFX Manager 3.1, and the normalized expression (ΔΔ*C_q_*) relative to the control was plotted in GraphPad Prism software (La Jolla, CA, USA):

GAPDH forward: ACAACTTTGGTATCGTGGAAGG
GAPDH reverse: GCCATCACGCCACAGTTTC
EB1/MAPRE1 forward: AAGCTAGAACACGAGTACATCCA
EB1/MAPRE1 reverse: AGTTTCTTGACCTTGTCTGGC
LIC2/DYNC1LI2 forward: GGCTAGTGTTTTACGTGAGCA
LIC2/DYNC1LI2 reverse: TGGGGAACCTTGACAACCTTC
HC1/DYNC1H1 forward: CCCCAAGATATGAAAGTGGCTG
HC1/DYNC1H1 reverse: CGATCCTCTCATCGTACCTCT

### Immunofluorescence microscopy

To visualize the localization of EB1 and LIC2 in 2D cultures, cells were plated on collagen I–coated 35-mm glass-bottom dishes. After 16 h, the cells were fixed with 3% paraformaldehyde for 10 min, permeabilized with 0.01% Triton X-100 for 10 min, and blocked with 1% bovine serum albumin for 1 h at room temperature. The cells were then stained for nuclear DNA (Hoechst 33342, 0.02 mg/ml), LIC2 (1:1000; kindly provided by Richard Vallee, Columbia University, New York, NY, USA), EB1 (1:1000; Cell Signaling Technology), and actin (Phalloidin, 1:40; Thermo Fisher Scientific). Fluorescent images for cells on 2D substrates were collected using a Cascade 1K CCD camera (Roper Scientific, Sarasota, FL, USA) mounted on a TE2000 Microscope (Nikon Instruments, Melville, NY, USA) with a ×60 oil immersion lens.

For cells embedded in 3D collagen I matrices, the incubation times for fixation and permeabilization were doubled to 20 min. Cells were then blocked with 1% bovine serum albumin for 2 h at room temperature. α-Tubulin antibody (1:500; Abcam, Cambridge, United Kingdom) was then added to the collagen I matrix and incubated at 4°C overnight. The next day, the gel was washed 3 times with PBS with 5 min between washes and incubated with mouse anti-human α-tubulin (1:200; Invitrogen), Phalloidin (1:40; Thermo Fisher Scientific), and Hoechst 33342 (0.02 mg/ml) for 2 h. The collagen I gel was washed 3 times with PBS and stored in PBS at 4°C. Imaging of matrix embedded cells was performed using a Nikon A1 Confocal Microscope.

### Embedding cells in 3D collagen I matrix and cell migration

As previously described, 2 mg/ml type I collagen gels were used for this study (12). After trypsinization, 18,000 cells were mixed in 1:1 ratio of cell culture medium and reconstitution buffer [0.2 M 4-(2-hydroxyethyl)-1-piperazineethanesulfonic acid (MilliporeSigma), 0.26 M sodium bicarbonate (NaHCO_3_) (MilliporeSigma), and water as solvent], and the appropriate amount of rat-tail collagen I (BD Biosciences) was added to achieve a final concentration of 2 mg/ml. Collagen I is solubilized in acetic acid, so the acid was quickly neutralized by adding a calculated amount of 1 N NaOH. Next, 500 μl of this final mixture was added per well of a 24-well dish, and the dish was incubated in a humidified incubator maintained at 37°C and 5% CO_2_. Fresh medium was added to the plate 2 h later, and the dish was put back in the incubator overnight.

Twenty-four hours later, images of cells were taken every 2 min for 16.5 h using an ORCA-AG 1K CCD camera (Hamamatsu Photonics, Shizuoka, Japan) mounted on a Nikon TE 2000 microscope base. Single cells were tracked using the template match algorithm in Metamorph imaging software. The *x*- and *y*- coordinates of cells obtained using Metamorph were processed using a MATLAB program to compute mean square displacements (MSDs) of cells using the following equation: MSD = [*x*(*t* + Δ*t*) − *x*(*t*)]^2^ + [*y*(*t* + Δ*t*) − *y*(*t*)]^2^.

### Protrusion topology

Time-lapsed movies were used to systematically count the number of protrusions present in individual cells in 3D matrix. We characterized the protrusion into zeroth-generation or mother protrusions, first-generation protrusions, and second-generation protrusions depending on their temporal location in the cell. Zeroth-generation protrusions started directly from the cell body (prolonging the nucleus) and were the first to emerge from the cell. Protrusions stemming from the zeroth-generation protrusions were termed first-generation protrusions, and protrusions that stemmed from the first-generation protrusions were termed second-generation protrusions. Zeroth-generation, first-generation, and second-generation protrusions for all tested conditions were counted and contrasted to determine whether protrusion number correlated with cell migration.

### Microtubule tracking

EFOR21 cells cultured in RPMI 1640 medium supplemented with 10% FBS were seeded in 3D collagen and on a 2D substrate and imaged using a Nikon A1 confocal microscope using the method introduced in Matov *et al.* (23). The number of comets imaged on each cell was analyzed using the U-Track software package (23, 24).

### RhoA activation and immunoblotting

HT1080 cells were plated on 2D substrates and inside 3D collagen I matrices and allowed to incubate for 48 h, which was the total duration of the motility experiments. A RhoA G-LISA kit (Cytoskeleton, Denver, CO, USA) was used to assess RhoA activity according to the manufacturer’s instructions. The amount of total RhoA was assessed using 12% SDS-PAGE and a more sensitive total RhoA ELISA (Cytoskeleton). Total RhoA blots used the same lysates used in the G-LISA assay. SDS-PAGE blots were probed with rabbit anti-RhoA antibody (Cell Signaling Technology) followed by anti-rabbit horseradish peroxidase antibody (Cell Signaling Technology).

### Cell diffusivity and anisotropic index

As described previously (25), we used the APRW model to break down cell trajectory coordinates into primary and secondary directions of migration. Next, the persistent time and speed in the primary axis (*P*_p_, *S*_p_) and the secondary axis (*P*_s_, *S*_s_) were computed by fitting MSDs in the primary (MSD_p_) and secondary axis (MSD_s_) according to the following equations:
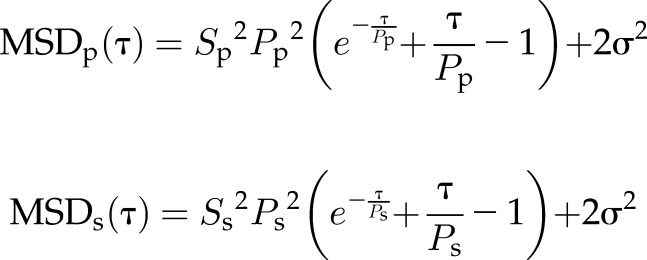
where τ represents time lag, and σ^2^ represents error in measurement of cell position. From the persistent time and speeds, we computed the diffusivity in the primary axis with the equation: 
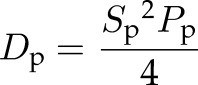
and the secondary axis with the equation:
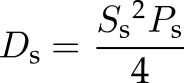
Isotropic was calculated as the ratio of *D*_p_ over *D*_s_.

### Vesicle trafficking

Vesicle-associated membrane protein 3 (VAMP3)-enhanced green fluorescent protein (EGFP) (plasmid 42310; Addgene) and LAMP1-mCherry (plasmid 45147; Addgene) were transfected into HT1080 cells using Lipofectamine 2000 (Thermo Fisher Scientific) according to product specifications. Six hours after transfection, the transfection reagent–containing medium was replaced with fresh medium. After 24 h, cells were trypsinized and plated on glass-bottom 24-well plates or embedded inside 3D collagen I gels as mentioned above. The next day (48 h after transfection), cells were imaged using a Nikon A1 confocal at 1 or 2 frames per second depending on the size of the scanned region. VAMP3-EGFP and LAMP1-mCherry dynamics were analyzed using the particle-tracking module in the U-Track software package (24, 26).

### Statistics

Statistical analyses were performed using GraphPad Prism software. Means ± sem is shown unless otherwise stated. One-way ANOVA and Student’s *t* test were performed wherever applicable to obtain statistical significance.

## RESULTS

### Microtubule dynamics is required for cell translocation in 3D matrix

Human fibrosarcoma cells (HT1080), a model system commonly used to study cell migration on 2D substrates and in 3D matrices (27–30), were either placed on 2D collagen I–coated substrates or fully embedded inside 3D collagen I matrices. Collagen I is the most abundant extracellular matrix protein in the stromal space near solid tumors (31, 32) and in connective tissues where fibrosarcoma tumors develop and disperse (33–35). To determine whether microtubule integrity and dynamics could play a role in 3D cell migration, cells were first treated with the microtubule-depolymerizing drug nocodazole and the microtubule-stabilizing drug taxol.

**Figure 1.**
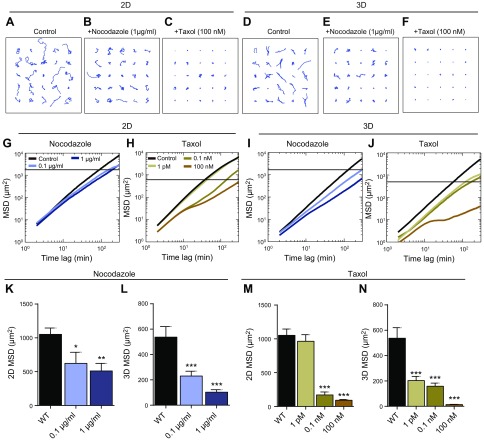
Microtubule dynamics mediates 3D cell migration. *A*–*F*). Typical trajectories of 25 individual control HT-1080 cells and cells treated with the microtubule-depolymerizing drug nocadozole and the microtubule-stabilizing drug taxol, migrating on collagen I–coated 2D substrates and inside 3D collagen I matrices. *G*, *H*). Population-averaged MSDs of control cells and cells treated with nocodazole (*G*) or taxol (*H*) migrating on 2D substrates. *I*, *J*). Population-averaged MSDs of control cells and cells treated with nocodazole (*I*) or taxol (*J*) migrating inside 3D collagen I matrix. *K*–*N*). MSDs of nocodazole- and taxol-treated cells migrating on 2D substrates (*K*, *M*) and in 3D matrix (*L*, *M*) evaluated at time scales of 1 h. For each condition, *n* = 3 biologic repeats; at least 60 cells were probed. *P < 0.05, **P < 0.01, ***P < 0.001.

Despite a presumed reduced accessibility of the drugs to the cells in the matrix, we observed a much more significant attenuation of cell migration inside the 3D matrix than for cells on 2D substrates when treated with the same concentration of nocodazole or taxol (**Fig. 1*A–N***). Trajectories of cells (*x*, *y* coordinates) were transformed into MSDs and evaluated (25, 36). On 2D substrates, the MSDs evaluated at 1 h were reduced by 41 (0.1 mg/ml) and 52% (1 mg/ml) for nocodazole-treated cells and by 8 (1 pM), 84 (0.1 nM), and 91% (100 nM) for taxol-treated cells compared with control cells (Fig. 1 *K*, *M*). In 3D matrices, the responsiveness of cells to nocodazole and taxol were further enhanced by 57 (0.1 mg/ml) and 81% (1 mg/ml) for nocodazole-treated cells and by 62 (1 pM), 70 (0.1 nM), and 97% (100 nM) for taxol-treated cells (Fig. 1*L*, *N*).

Together these results demonstrate that microtubule dynamics play a role in controlling cell speed in the pathologically relevant case of a 3D matrix, significantly more so than in the conventional 2D case. Counterintuitively, taxol treatment of fibrosarcoma cells is significantly more effective at affecting and blocking cell migration in 3D matrix than on 2D substrate.

### Cells in 3D matrix form dendritic protrusions made of an inner ring of microtubules and an outer ring of F-actin bundles

**Figure 2.**
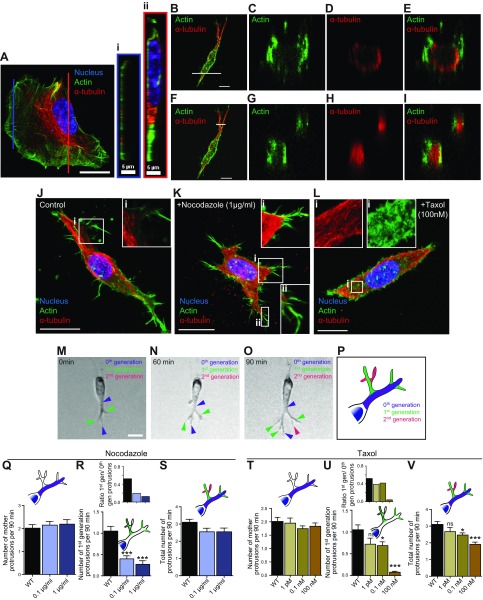
Microtubule dynamics promotes protrusion branching in 3D matrix. *A*–*I*). Actin filament and microtubule organization in control HT1080 cells growing on 2D substrates (*A*) and inside 3D matrix (*B*–*I*). Cross-sections through the lamellipodium (right panel *i*, orange border) and the perinuclear cell body (right panel *ii*, red border). Cells were stained with DAPI (nuclear DNA) and antibodies against microtubule (red) and actin filament (green). *J*–*L*). Immunofluorescent images of control cells (*J*) and cells treated with nocodazole (*K*) and taxol (*L*) embedded in 3D collagen I matrices. Insets: Daughter protrusions arising from the mother protrusions that prolong the nucleus are filled with microtubules in their lumen and F-actin at the periphery (*J*); nocodazole treatment increases the number of filopodial-like protrusions (*K*); taxol treatment gives rise to short, hairy actin protrusion throughout the cell (*L*). Cells were stained with DAPI (nuclear DNA) and antibodies against microtubule (red) and actin filament (green); images were obtained by immunofluorescence microscopy. *M*–*P*). Active formation of pseudopodial protrusions by a cell embedded inside a 3D matrix (purple arrowheads, zeroth-generation or mother protrusions that stems directly from the cell body; green arrowheads, first-generation protrusions that start from the zeroth-generation protrusions; magenta arrowheads, second-generation protrusions that start from the first-generation protrusions). Schematic showing zeroth-, first-, and second-generation protrusions in a cell (*P*). *Q*–*V*). Total number of mother protrusions (zeroth-generation protrusions) generated per 90 min per cell in nocodazole- and taxol-treated cells (*Q*, *T*). Number of first-generation protrusions generated per 90 min per cell (*R*, *U*). Insets: Number of first-generation protrusions per mother protrusion (degree of branching) (*R*, *U*); total number of protrusions generated per 90 min per cell (*S*, *V*). For all panels, cells were monitored for 16.5 h. For each condition, *n* = 3; at least 40 cells were probed for protrusion topology analysis. WT, wild type. *P < 0.05, ***P < 0.001. Scale bars, 20 µm.

To assess the different migratory phenotypes observed in the nocodazole-treated and taxol-treated cells in 2- and 3D, we assessed the morphology and the organization of microtubules and actin filaments for cells in 3D matrix and on substrates. Rather than a fan-shaped morphology with a wide lamellipodium and thin terminal filopodial protrusions at the leading edge of cells on 2D substrates (**Fig. 2*A***), cells embedded in 3D matrix displayed highly branched dendritic protrusions (Fig. 2*B*, *J*) [see also Giri *et al.* (5)]. These branched protrusions contained both microtubules and F-actin (Fig. 2*B–I*). Cross-sections through the perinuclear cell body and through protrusions far from the nucleus of cells in matrix showed circumferential arrangement of F-actin and microtubule bundles, with actin filaments forming an outer ring of discrete F-actin bundles and microtubules forming an inner ring or core (Fig. 2*B–I*). Thick protrusions (*e.g.*, protrusions prolonging the cell body; Fig. 2*B–E*) showed an inner lumen with few microtubule and actin stains in the center and concentric rings of microtubules and F-actin. For thinner protrusions (*e.g.*, Fig. 2*F–I*), microtubules formed a core in the center of the protrusions with no lumen and an outer ring of F-actin. In contrast to the 3D case, the cross-section of lamellipodial protrusions in flattened cells on substrates typically showed no dense arrangement of microtubules in the lamellipodium (Fig. 2*A*, profile *i*); rather, higher microtubule density was observed in the perinuclear region in the direction of migration (Fig. 2*A*, profile *ii*). Moreover, filopodia did not contain microtubules, as extensively shown previously (Fig. 2*A*). The high content of microtubules in protrusions of matrix-embedded cells (Fig. 2*B*) compared with the low content in protrusions of cells on 2D substrates (in the lamellipodium and filopodia; Fig. 2*A*) is consistent with the much higher sensitivity of cells to microtubule drugs in the 3D case compared with the conventional 2D case (Fig. 1).

### Microtubule dynamics mediates dendritic protrusions

Recent studies have shown a strong correlation between 3D cell migration speed and protrusion activity, defined here as the number of protrusions generated per unit time, not the length or lifetime of protrusions (5, 11, 12). Therefore, we hypothesized and verified that pharmacological manipulation of microtubules using nocodazole and taxol would reduce protrusion activity because these treatments reduce cell speed in 3D matrices (Fig. 2*M–V*).

Following the nomenclature introduced previously (5), we refer to the pseudopodial protrusions directly prolonging the nucleus as zeroth-generation protrusions or mother protrusions (Fig. 2*M–O*, blue arrowheads). These zeroth-generation protrusions branched further into first-generation protrusions (Fig 2*M–O*, green arrowheads), which further branched into second-generation protrusions (Fig 2*M–O*, magenta arrowheads). The first-generation and second-generation protrusions are collectively referred to as daughter protrusions [green and magenta branches (Fig. 2*P*); see Materials and Methods and Giri *et al.* (5) for more details about the use of movies to determine the generation number of protrusions]. Time-lapse movies were used to classify protrusions based on the temporal order in which protrusions appeared and disappeared. We observed that the formation of daughter protrusions was drastically reduced in both nocodazole- and taxol-treated cells (Fig. 2*R*, *U*), but the number of mother protrusions was not significantly changed in these cells (Fig. 2*Q*, *T*). In matrix-embedded nocodazole-treated cells, the rates of formation of first-generation protrusions decreased ∼3-fold (0.1 µg/ml) and ∼4-fold (1 µg/ml), and the rates of formation of second-generation protrusions decreased 9-fold (0.1 µg/ml). No second-generation protrusions were detected at 1 µg/ml compared with control cells (Fig. 2*R* and Supplemental Fig. 2*B*).

Similarly, in taxol-treated cells, the rates of formation of first-generation protrusions decreased ∼1.5-fold (1 pM), ∼1.5-fold (0.1 nM), and ∼14-fold (100 nM); rates of formation of second-generation protrusions decreased ∼1.4-fold (1 pM) and 1.7-fold (0.1 nM); and no second-generation protrusion was detected at 100 nM compared with control cells (Fig. 2*U* and Supplemental Fig. 2*A*). Accordingly, the degrees of protrusive branching off mother protrusions were drastically reduced in nocodazole- and taxol-treated cells (Fig. 2*R*, *U*, insets). We note that the treatment of cells with nocodazole and taxol increased somewhat the formation of short filopodia-like protrusions in 3D (Fig. 2*K*, *L*), similar in length, thickness, and actin content as filopodia at the leading edge of cells on flat substrates but distinct in morphology from the microtubule-containing pseudopodial protrusions described above. Because these treatments abrogate cell motility in 3D, these small and thin protrusions do not seem to play a significant role in 3D migration.

These findings suggest that microtubule dynamics promote 3D cell migration by increasing the degree of branching of microtubule-filled protrusions presented by cells in 3D matrix, a type of protrusion that is absent from cells flattened by 2D substrates.

### EB1 promotes 3D cell migration by mediating high protrusion activity and branching

Stabilizing microtubules with taxol treatment does not stabilize pseudopodial daughter protrusions but rather eliminates them (Fig. 2*U*). Hence, because the above results suggested that microtubules did not play a structural role in 3D migration *per se*, we hypothesized that microtubules instead had a regulatory role through assembly/disassembly dynamics. To determine the mechanism by which microtubule dynamics induce effective cell migration in 3D matrix and modulate protrusion generation (Figs. 1 and 2), we asked whether disruption of microtubule dynamics by specific depletion of the major microtubule-tip binding protein (Supplemental Fig. 1*A*), which regulates microtubule stability at least for cells on 2D dishes (13, 14, 37–40), would reduce 3D cell migration.

**Figure 3.**
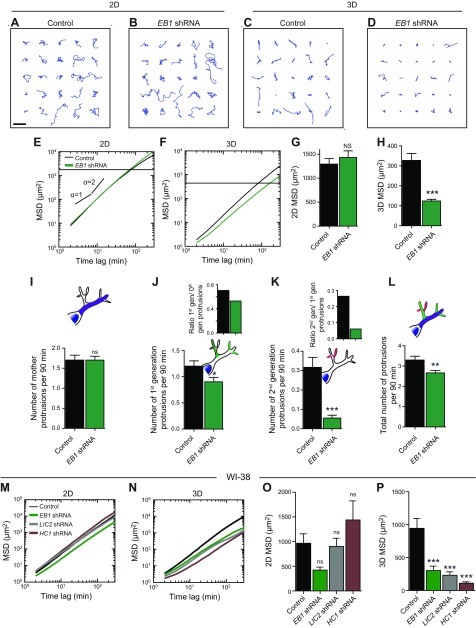
The distinct role of EB1 in 3D cell migration. *A*–*D*). Typical trajectories of 25 individual control and EB1*-*depleted cells migrating on collagen I–coated 2D substrates and inside 3D collagen I matrices. Scale bar, 200 µm. *E*, *F*). shRNA-mediated depletion of EB1 has an inhibitory effect on cell migration in cells migrating in collagen I matrices (*F*) but has no significant effect on cell migration on substrates (*E*). *G*, *H*). Regulation of migration for cells on 2D substrates and embedded in 3D collagen I matrices by EB1. MSDs were evaluated at a time scale of 1 h. *I*). Total number of mother protrusions (zeroth-generation protrusions) generated per 90 min per cell. *J*, *K*). Number of first-generation protrusions (*J*) and second-generation protrusions (*K*) generated per 90 min per cell. Insets: Number of first-generation protrusions per mother protrusion (*J*); number of second-generation protrusions per first-generation protrusion (*K*). *L*). Total number of protrusions generated per 90 min per cell. For all panels, cells were monitored for 16.5 h. *M*, *N*). MSD plots for WI-38 cells depleted of EB1, LIC2, and HC1 moving on 2D substrates (*M*) and inside collagen I matrix (*N*). *O*, *P*). MSDs for WI-38 cells evaluated at a time scale of 1 h for cells on 2D substrates (*O*) and inside 3D matrix (*P*). For each condition, *n* = 3; at least 60 cells were probed for cell migration analysis, and at least 40 cells were probed for protrusion topology analysis. Ns, not significant. **P < 0.01, ***P < 0.001.

Visual inspection of trajectories of cells on substrates readily suggested no qualitative difference in 2D migration between control cells (transfected with scrambled shRNA construct) and cells depleted of EB1 (**Fig. 3*A*, *B***). Indeed, the MSDs of EB1-depleted cells and control cells overlapped (Fig. 3*E*). The difference in MSD values between control and EB1-depleted cells on substrates was miniscule (Fig. 3*G*). In contrast, EB1-depleted cells migrating inside 3D collagen I matrices had much tighter trajectories compared with control cells (Fig. 3*C*, *D*, *F*). In 3D matrices, MSD evaluated at 1 h were reduced by ∼62% compared with control cells (Fig. 3*H* and Supplemental Fig. 1*M*). These results suggest that, although the microtubule-tip binding protein EB1 is dispensable in 2D cell migration, it is an integral contributor to 3D cell migration.

We then speculated that EB1 promoted 3D cell migration by mediating a high degree of protrusive branching of cells in 3D matrices. Indeed, after EB1 depletion, the rates of formation of first- and second-generation protrusions decreased by ∼31 and 84% respectively, without affecting mother protrusions (Fig. 3*I–L*). Accordingly, the degrees of protrusive branching of mother and first-generation protrusions were significantly reduced (Fig. 3*J*, *K*, insets).

To further verify the effect of EB1 on 3D cell migration, we depleted EB1 in WI-38 cells, a lung fibroblast cell line that is widely used to study cell migration. As expected, EB1 depletion in WI-38 cells did not significantly alter cell migration on 2D substrates (Fig. 3*M*, *O*), but it drastically reduced cell migration in a 3D matrix (Fig. 3*N*, *P*). Furthermore, using EB1-EGFP comet detection and tracking demonstrated that taxol treatments drastically altered microtubule dynamics for cells in a 3D matrix when compared with the 2D condition. The total number of EB1 comets per cell significantly decreases when cells in 3D were treated with 10 pM of taxol. No comets were observed at a dose of 1 nM. In 2D, EB1 comets were observed, with no significant differences between the control condition and cells treated with 1 nM of taxol (Supplemental Fig. 1*G*).

Considering these observations and the high sensitivity of 3D cell migration to taxol and nocadozole treatments, our results suggest a 3D-specific role for EB1, where it is a major regulator of 3D cell migration, through the promotion of the formation of dendritic protrusions in 3D.

### LIC2 and HC1 domains of cytoplasmic dynein mediate 3D cell migration by promoting high protrusion activity and branching

Next we asked if selective manipulation of other microtubule-binding proteins would have a similar selective effect on 3D cell migration. Because protrusion generation, which is critical to 3D cell migration, could be modulated by microtubule-based mechanisms distinct from microtubule dynamics, such as vesicular trafficking, we hypothesized that minus end-directed motor protein dynein could play a role in the regulation of 3D cell migration. Using shRNA-mediated knockdowns, we created cell lines depleted of LIC2, a noncatalytic subunit of dynein, and HC1, a catalytic subunit of dynein.

**Figure 4.**
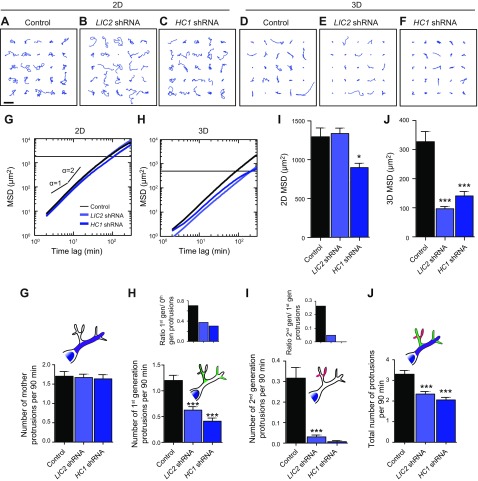
The distinct role of LIC2 and HC1 in 3D cell migration. *A*–*F*). Typical trajectories of 25 individual control, LIC2*-*, and HC1-depleted cells migrating on collagen I–coated 2D substrates and inside 3D collagen I matrices. Scale bar, 200 µm. *G*, *H*). shRNA-mediated depletion of LIC2 has an inhibitory effect on cell migration in cells migrating in collagen I matrices (*H*) but has no significant effect on cell migration on substrates (*G*). *I*, *J*). Regulation of migration for cells on 2D substrates (*I*) and in 3D collagen I matrices (*J*) by LIC2. MSDs were evaluated at a time scale of 1 h (*I*, *J*). *K*). Total number of mother protrusions (zeroth-generation protrusions) generated per 90 min per cell. *L*, *M*). Number of first-generation protrusions (*L*) and second-generation protrusions (*M*) generated per 90 min per cell. Insets: Number of first-generation protrusions per mother protrusion (*L*); number of second-generation protrusions per first-generation protrusion (*M*). *N*). Total number of protrusions generated per 90 min per cell. For all panels, cells were monitored for 16.5 h. For each condition, *n* = 3; at least 60 cells were probed. *P < 0.05, ***P < 0.001.

On 2D substrates, cells depleted of LIC2 migrated as rapidly as control cells (**Fig. 4*A*, *B*, *G*, *I***), whereas cells depleted of HC1 were slower than control cells (Fig. 4*C*, *G*, *I*). However, when LIC2- and HC1-depleted cells were placed inside 3D collagen matrices, the trajectories of these cells were much tighter and their MSDs were much smaller compared with control cells (Fig. 4*D–F*, *H*, *J*). To verify if cell migration was again mediated by changes in the number of daughter protrusions, we quantified the topology of the protrusions in LIC2- and HC1-depleted cells. LIC2 and HC1 depletion significantly reduced the number of daughter protrusions without changing the number of mother protrusions (Fig. 4*K–N*). The rates of formation of first-generation protrusions for LIC2- and HC1-depleted cells decreased 2- and 3-fold, respectively (Fig. 4*L*). The rates of formation of second-generation protrusions for LIC2- and HC1-depleted cells decreased 10- and 44-fold, respectively (Fig. 4*M*). Accordingly, the degree of branching of first-generation protrusions (off mother protrusions) and second-generation protrusions (off first-generation protrusions) were significantly reduced in LIC2- and HC1-depleted cells compared with control cells (Fig. 4*L*, *M*, insets).

We verified the above results using WI-38 human lung fibroblasts. Depletion of the LIC2 and HC1 domains of dynein had no statistically significant effect on 2D cell migration (Fig. 3*M*, *O*) but greatly reduced cell migration in 3D matrix (Fig. 3*N*, *P*).

These results indicate that cytoplasmic dynein plays a significant role in 3D cell migration by tightly regulating the degree of branching of 3D-specific dendritic protrusions through its domains LIC2 and HC1.

### EB1 and dynein mediate the directionality of cell migration inside 3D matrix

Previous studies have shown that cell migration in 3D matrix does not follow conventional random-walk statistics but rather an anisotropic random walk (25). To study if EB1 and dynein mediated not only the speed of migration but also the directionality of cell migration, we fit experimental MSDs to the APRW model introduced in Wu *et al.* (25). This model decomposes cell velocities into primary and secondary directions of migration of individual cells and provides persistent time and speed along the primary axis (*P*_p_, *S*_p_) and the secondary axis (*P*_s_, *S*_s_) of migration. From these migratory descriptors, we computed the diffusivity along the primary axis (*D*_p_) and secondary axis (*D*_s_) as *D*_p_ = *S*_p_^2^*P*_p_/4 and *D*_s_ = *S*_s_^2^*P*_s_/4, respectively (Supplemental Fig. 4*A*–*L*). We also computed the anisotropic index as the ratio of *D*_p_ over *D*_s_, a quantity that measures directionality of migration.

**Figure 5.**
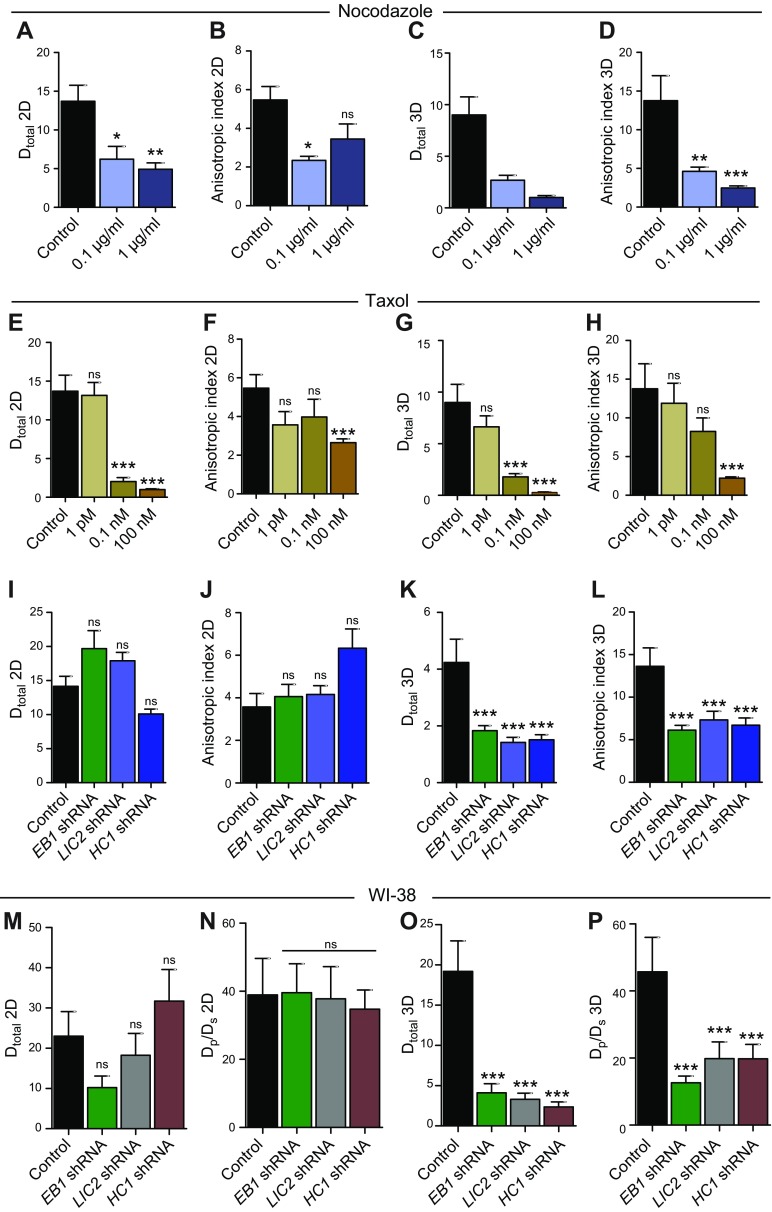
Cell migration in 3D matrix is anisotropic. *A*–*D*). Total diffusivity and anisotropic index for nocodazole-treated cells on 2D (*A*, *B*) and in 3D matrix (*C*, *D*). *E*–*H*). Total diffusivity and anisotropic index for nocodazole-treated cells on 2D (*E*, *F*) and in 3D matrix (*G*, *H*). *I*–*L*). Total diffusivity and anisotropic index for EB1-, LIC2-, and HC1-depleted cells on 2D (*I*, *J*) and in 3D matrix (*K*, *L*). *M*–*P*). Total diffusivity and anisotropic index for EB1-, LIC2-, and HC1-depleted WI-38 cells on 2D (*M*, *N*) and in 3D matrix (*O*, *P*). Ns, not significant. *P < 0.05, **P < 0.01, ***P < 0.001.

On 2D substrates, nocodazole and taxol treatments caused a dose-dependent decrease in the total diffusivity (*D*_total_ = *D*_p_ + *D*_s_) of cells in 3D matrix, which means that nocodazole- and taxol-treated cells migrated more slowly than control cells (**Fig. 5*A*, *E***). In 3D matrix, nocodazole and taxol treatments also caused a decrease in total diffusivity of the cells; however, the effects were much more pronounced compared with the 2D case (Fig. 5*C*, *G*). On both 2D substrates and inside 3D matrices, the anisotropic index for nocodazole- and taxol-treated cells was smaller compared with the control cells, which implies that microtubules maintain directional cell migration (Fig. 5*B*, *D*, *F*, *H*).

On 2D substrates, the total diffusivity of EB1-, LIC2-, and HC1-depleted cells was similar to that of control cells, whereas the diffusivity of these cells in 3D matrix was significantly smaller compared with control cells (Fig. 5*I*, *K*). This result obtained from the APRW model fits is in agreement with our experimental MSD values (Figs. *3G*, *H* and 4*I*, *J*). On 2D substrates, the anisotropic indices for EB1-, LIC2-, and HC1-depleted cells were comparable to that of control cells, whereas for cells inside 3D matrices, the anisotropic indices for EB1, LIC2, and HC1 depleted cells were reduced by 52, 42, and 47%, respectively (Fig. 5*J*, *L*).

Analysis of diffusivity and anisotropic index in WI-38 cells showed similar results. On 2D substrates, the total diffusivity and anisotropic index for EB1-, LIC2-, and HC1-depleted cells were not significantly different from control cells (Fig. 5*M*, *N*). In 3D matrix, the *D*_total_ and anisotropic index for EB1-, LIC2-, and HC1-depleted cells were significantly reduced compared with control cells (Fig. 5*O*, *P*).

### EB1 and dynein promote 3D migration by inducing high microtubule dynamics in protrusions

**Figure 6.**
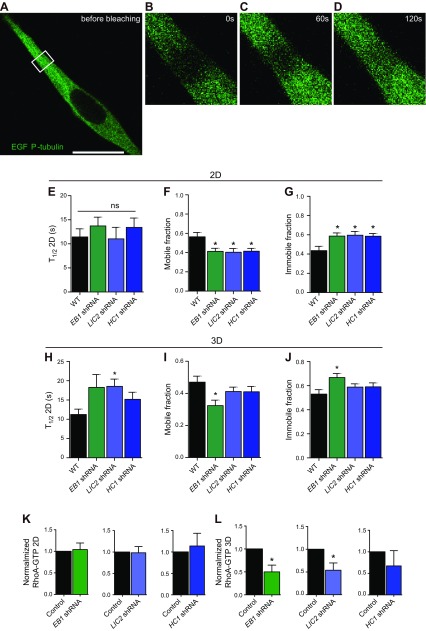
LIC2 and EB1 promote fast microtubule dynamics in pseudopodial protrusions of cells in 3D matrix *via* RhoA. *A*–*D*). FRAP images of cells embedded inside 3D collagen I matrices. Time of image acquisition is mentioned in the images. *E*–*J*). Half-life of fluorescence recovery, mobile fraction, and immobile fraction of α-tubulin GFP for control and for EB1-, LIC2-, and HC1-depleted cells on 2D substrates (*E*–*G*) and inside a 3D matrix (*H*–*J*). *K*, *L*). RhoA-GTP. Ns, not significant. *P < 0.05.

Given the above results, we hypothesized that EB1, and to a lesser extent dynein, regulated 3D migration by modulating microtubule dynamics. Microtubule dynamics were assessed by fluorescence recovery after bleaching (FRAP) of cells transfected with α-tubulin EGFP (13, 37). Cells were placed inside a 3D collagen I matrix and monitored (**Fig. 6*A–D***). FRAP analysis revealed that microtubule dynamics were much slower in matrix-embedded cells than in cells on 2D substrates, as measured by a shorter halftime for recovery (*T*_1/2_) and lower recovered fraction in control cells and cells depleted of LIC2 or EB1 (Fig. 6*E–J*). Although EB1 and dynein played a minor role in microtubule dynamics in fibrosarcoma cells on 2D substrates (Fig. 6*E*), EB1 and dynein promoted fast microtubule dynamics for cells in 3D matrix (Fig. 6*H*).

Because microtubules and microtubule-associated proteins play a role in vesicular trafficking, at least for cells on 2D substrates, and because such trafficking may modulate cell migration, we further determined whether EB1 and dynein regulated vesicular trafficking along microtubules by transfecting live cells with VAMP3-EGFP and LAMP1-mcherry constructs (41). Using time-dependent confocal microscopy, we tracked the speed of vesicles inside live cells and found that these proteins played no significant role in the trafficking of VAMP3- and LAMP1-tagged vesicles (Supplemental Fig. 3*A*–*D*).

EB1, LIC2, and HC1 appear to play no significant function in 2D migration but are critically required for 3D migration. Members of the Rho family of GTPases are key regulators of actin and microtubule dynamics (1, 42). In particular, a large body of work has highlighted functional interactions between microtubules and RhoA GTPase, where its activation is mediated by guanine nucleotide exchange factors (43, 44). Guanine exchange factors are sequestered along microtubule fibers (42, 43, 45). Because the loss of microtubule dynamics or its associating proteins would result in the disruption of binding and transport of these proteins, we hypothesized that the reduction in microtubule dynamics would result in a decrease in RhoA activity. We quantified RhoA activity of control cells and cells depleted of EB1, LIC2, and HC1 in 2- and 3D settings. In 2D, EB1-, LIC2-, and HC1-depleted cells displayed no significant change in RhoA activity compared with control cells, whereas in 3D, these cell lines collectively displayed a decrease in RhoA activity (Fig. 6*K*, *L*).

Together these results suggest that EB1 and dynein mediate migration in 3D by promoting microtubule dynamics, through the activity of RhoA, in microtubule-rich pseudopodial dendritic protrusions that drive cell migration in 3D.

## DISCUSSION

Our findings indicate that microtubule dynamics are critical to migration in the pathologically relevant case of 3D collagen matrices but not on 2D collagen–coated substrates because the drivers of 3D cell migration (*i.e.*, dendritic pseudopodial protrusions) are filled with dynamic microtubules that critically rely on microtubule-tip protein EB1 and microtubule-associated motor protein dynein (LIC2 and HC1) to branch out into the matrix. Furthermore, our findings suggest that microtubules play structural and regulatory roles in effective 3D migration. Quantitative live-cell measurements show that microtubule turnover dynamics (Fig. 5)—not vesicular trafficking along microtubules (Supplemental Fig. 3) and intrinsic microtubule organization (Fig. 2)—account for the modulation of 3D cell migration by microtubules and associated proteins.

The differences in migration observed in 2- and 3D settings provide evidence that changes in extracellular and environmental cues may be responsible for the different phenotypes observed. We observed that in 2D settings, RhoA activity remained unchanged when microtubule-associated proteins when depleted, resulting in no significant change in cell migration. However, when the same proteins were depleted in 3D settings, we observed that RhoA activity was decreased. This suggests that microtubule-associated proteins EB1, LIC2, and HC1 are required to establish RhoA activity in 3D but not in 2D. This may be a result of the reduction of binding or transport of guanine nucleotide exchange factors due to either the decrease in microtubule stability or the loss of association with microtubule-associating proteins (38, 46–49). In light of these observations, further investigation is necessary to compare the activity in other Rho-family GTPases in these 2 environments, specifically Rac1 and Cdc42, which have been implicated to play a role in cell migration (50–55). Their influence on microtubule dynamics in 3D migration has yet to be investigated.

Results of this study also suggest that one of the main reasons for this lack of functional relationship between 2D and 3D migration is that the major cytoskeletal filaments F-actin and microtubules are organized in matrix-embedded cells that are fundamentally differently from their 2D counterparts (Fig. 2). Actin filaments and microtubules form concentric rings of bundles oriented along the long axis of the protrusions, with F-actin forming the outer ring (presumably attributed to membrane-binding proteins, such as members of the ERM family of proteins) and microtubules forming the inner ring. Strikingly, inside protrusions, F-actin and microtubule barely mix but form a seemingly tight interface. Indeed, in thick protrusions, the inner ring of microtubules is favorably connected to the outer ring of F-actin rather than filling up the core of the protrusions.

This and recent work (5, 11, 12) indicate that mother protrusions and the daughter protrusions that dendritically grow from them are regulated by distinct families of molecules. The degree of branching from mother protrusions (*i.e.*, the number of daughter protrusions per mother protrusions) is specifically regulated by microtubule dynamics through microtubule-associated proteins EB1 and dynein (LIC2 and HC1). These proteins are part of an increasing family of proteins that specifically regulate branching off from mother protrusions. In addition to EB1 and dynein, the degree of branching from mother protrusions is specifically regulated by focal adhesion proteins and F-actin regulators vasodilator-stimulated phosphoprotein and zyxin (5), F-actin nucleator Arp2/3 complex, Arp2/3-complex regulators/activators N-Wasp, Cortactin, Wave 1, and small GTPase Cdc42 (5). These proteins do not mediate the formation of mother protrusions. In contrast, the formation of mother protrusions is specifically regulated by focal adhesion proteins focal adhesion kinase, talin, and p130Cas (5); these proteins do not modulate side branching from mother protrusion. The results of this study add to the increasing body of evidence that only molecular mediators of protrusive branching regulate 3D cell migration and that the degree of branching from mother protrusions, and not the ability of cells to form mother protrusions themselves *per se*, reliably predicts cell speed in 3D matrices (see global correlations in Supplemental Fig. 2*C*–*G*).

A possible clinical consequence of our results is that the microtubule-stabilizing drug paclitaxel, commonly used for tumor shrinkage in a wide range of human cancers (*e.g.*, paclitaxel treatment is standard of care for breast, ovarian cancer, and fibroblastic tumors), is significantly more effective at arresting cell migration in 3D matrix than on 2D substrates. This observation emphasizes the necessity of adopting a more robust testing platform that incorporates screening pharmacological agents in 3D matrices instead of conventional 2D substrates to potentially predict efficacy and specificity.

## Supplementary Material

This article includes supplemental data. Please visit *http://www.fasebj.org* to obtain this information.

## Abbreviations

2D: 2-dimensional
3D: 3-dimensional
APRW: anisotropic persistent random walk
EB1: end-binding 1
EGFP: enhanced green fluorescent protein
FBS: fetal bovine serum
FRAP: fluorescence recovery after bleaching
HC1: heavy chain 1
LIC2: light intermediate chain 2
MSD: mean squared displacement
shRNA: short hairpin RNA
VAMP3: vesicle-associated membrane protein 3
