# Supplementary material for: EB1 and cytoplasmic dynein mediate protrusion dynamics for efficient 3-dimensional cell migration
Source: FASEB J. 2017 Nov 2;32(3):1207–21. doi: 10.1096/fj.201700444RR (PMC5893312; doi:10.1096/fj.201700444RR)
Supplement: Supplementary file 1 [file fj.201700444RR.sd1.docx]

**Supplementary Figure Captions**

**Supplementary Figure 1. A-C.** Western blots of control and cells depleted of EB1 (A), LIC2 (B), and HC1 (C). Two constructs were assessed per protein. **D-F.** Decreased expression of EB1, LIC2, and HC1 in shRNA mediated knockdowns as measured by qRTPCR **G.** Total number of EB1 comets per cell observed in 2D and 3D environments when treated with varying doses of Taxol. **H-K.** Population-averaged mean squared displacements (MSDs) of control cells and cells treated with Taxol (H and I) and Nocodazole (J and K) at the very beginning of the observation period (4 minutes after treatment) in 2D and 3D environments.

**Supplementary Figure 2. A and B.** Total number of second generation protrusions generated per cell per 90 min by Taxol (A) and Nocodazole (B) treated cells. **C-G.** Correlation analysis that indicates there is no correlation between cell motility (measured as MSDs) in 2D and 3D environments (C), strong correlation between 3D cell motility and daughter protrusions (D and E), and no correlation between 3D cell motility and mother protrusions (F and G). Circled data points are new data from this work; other data is taken from ref. [^5^](#_ENREF_5)^,^[^8^](#_ENREF_8)^,^[^9^](#_ENREF_9).

**Supplementary Figure 3. A and B.** Migration speed of LAMP1-GFP tagged vesicles moving inside live cells growing on 2D substrates (A) or inside 3D collagen I matrix (B). **C and D.** Persistence of vesicle movement defined as displacement divided by total length of cells growing on 2D substrates (C) and inside 3D matrix (D). **E and F.** Western blot of RhoA from control and cells depleted of EB1, LIC2, and HC1 that were cultured in a 2D (E) and 3D (F) environment.

**Supplementary Figure 4. A-D.** Diffusivity of the cells treated with nocodazole in the primary and secondary axis of migration on 2D (A and B) and in 3D matrix (C and D). **E-H.** Diffusivity of the cells treated with taxol in the primary and secondary axis of migration on 2D (E and F) and in 3D matrix (G and H). **I-L.** Diffusivity of the Control, EB1-, LIC2-, and HC1- depleted cells in the primary and secondary axis of migration on 2D (I and J) and in 3D matrix (K and L).
