# Supplementary figures and images for: EB1 and cytoplasmic dynein mediate protrusion dynamics for efficient 3-dimensional cell migration
Source: FASEB J. 2017 Nov 2;32(3):1207–21. doi: 10.1096/fj.201700444RR (PMC5893312; doi:10.1096/fj.201700444RR)

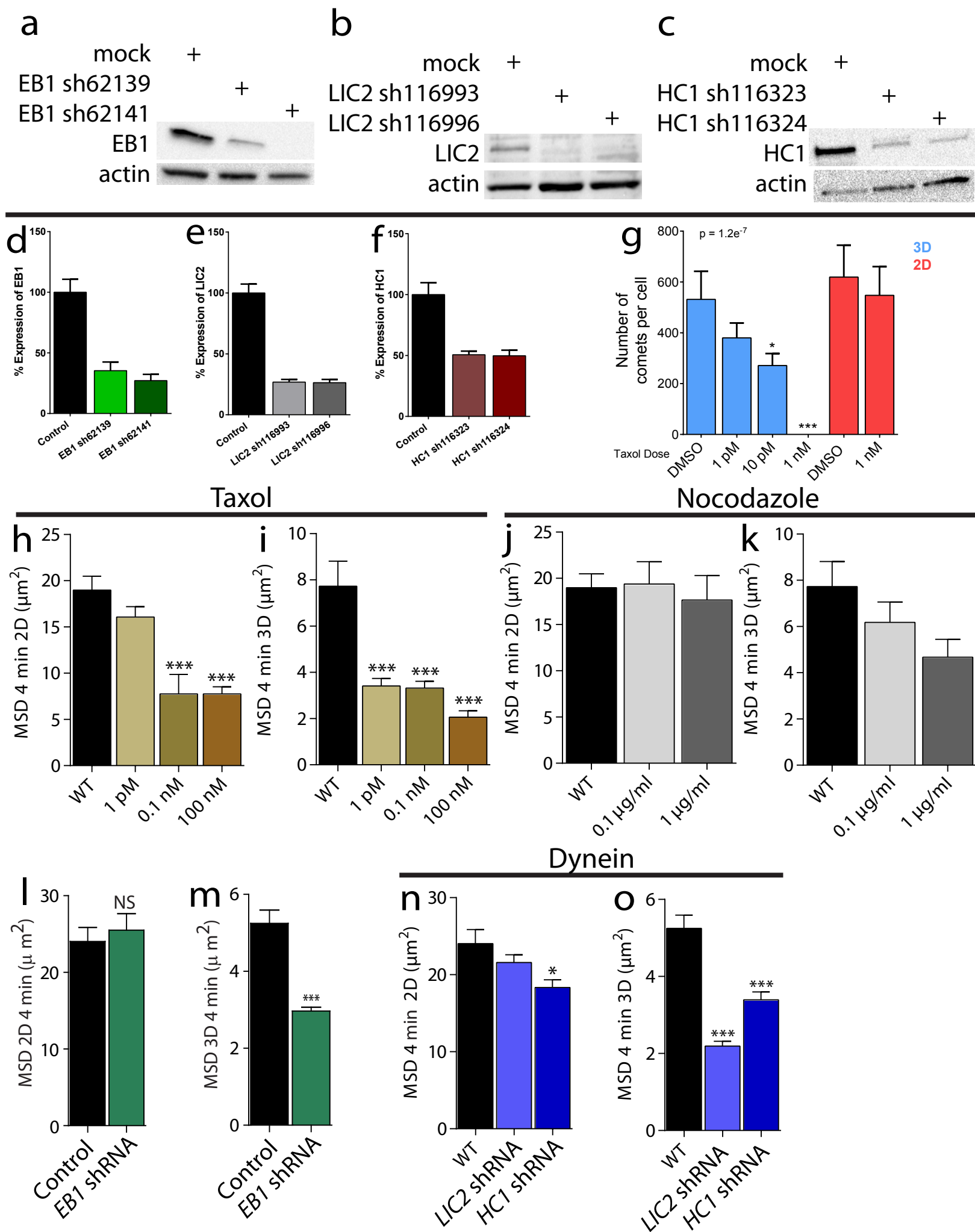

Supplemental figure 1 - Jayatilaka et al.

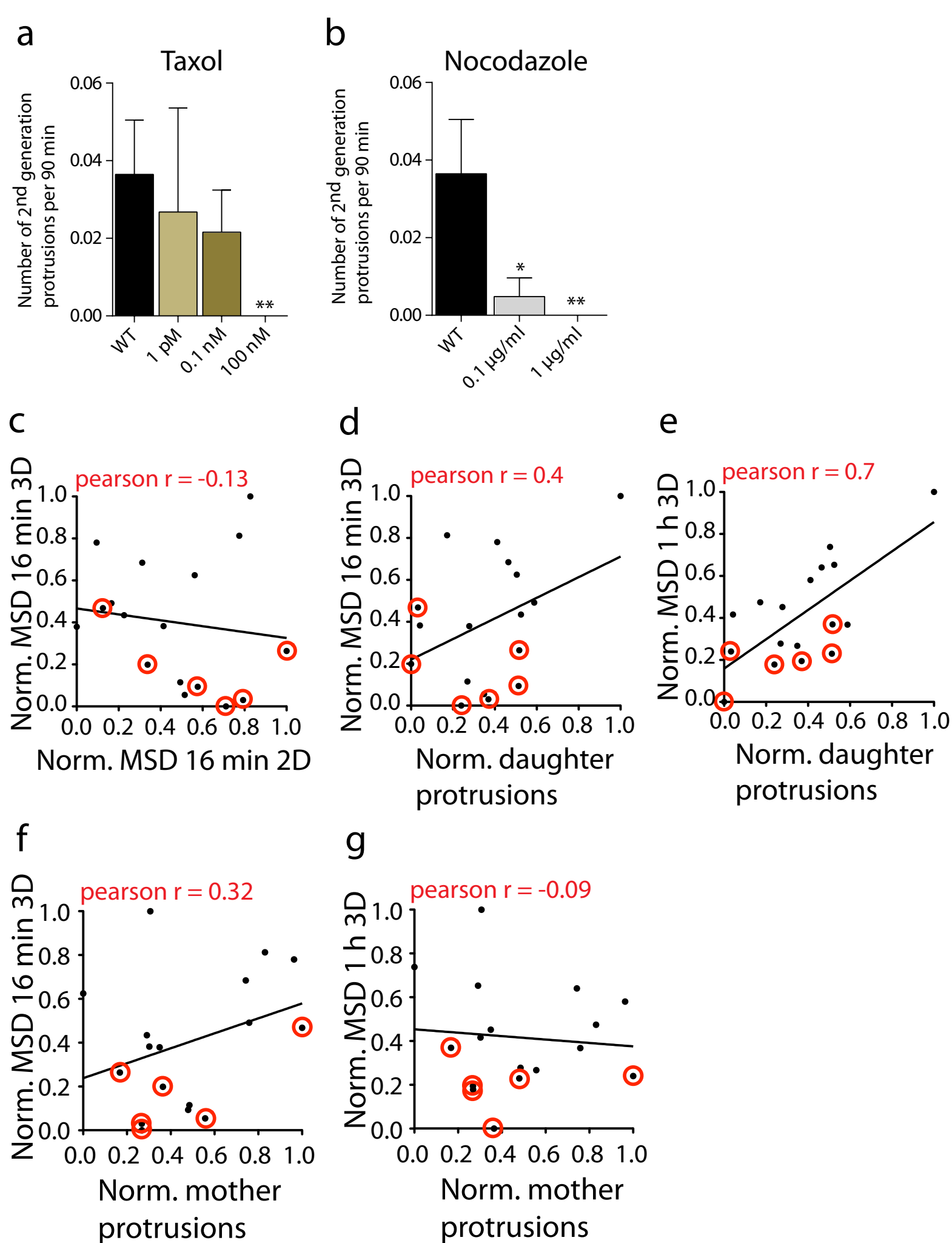

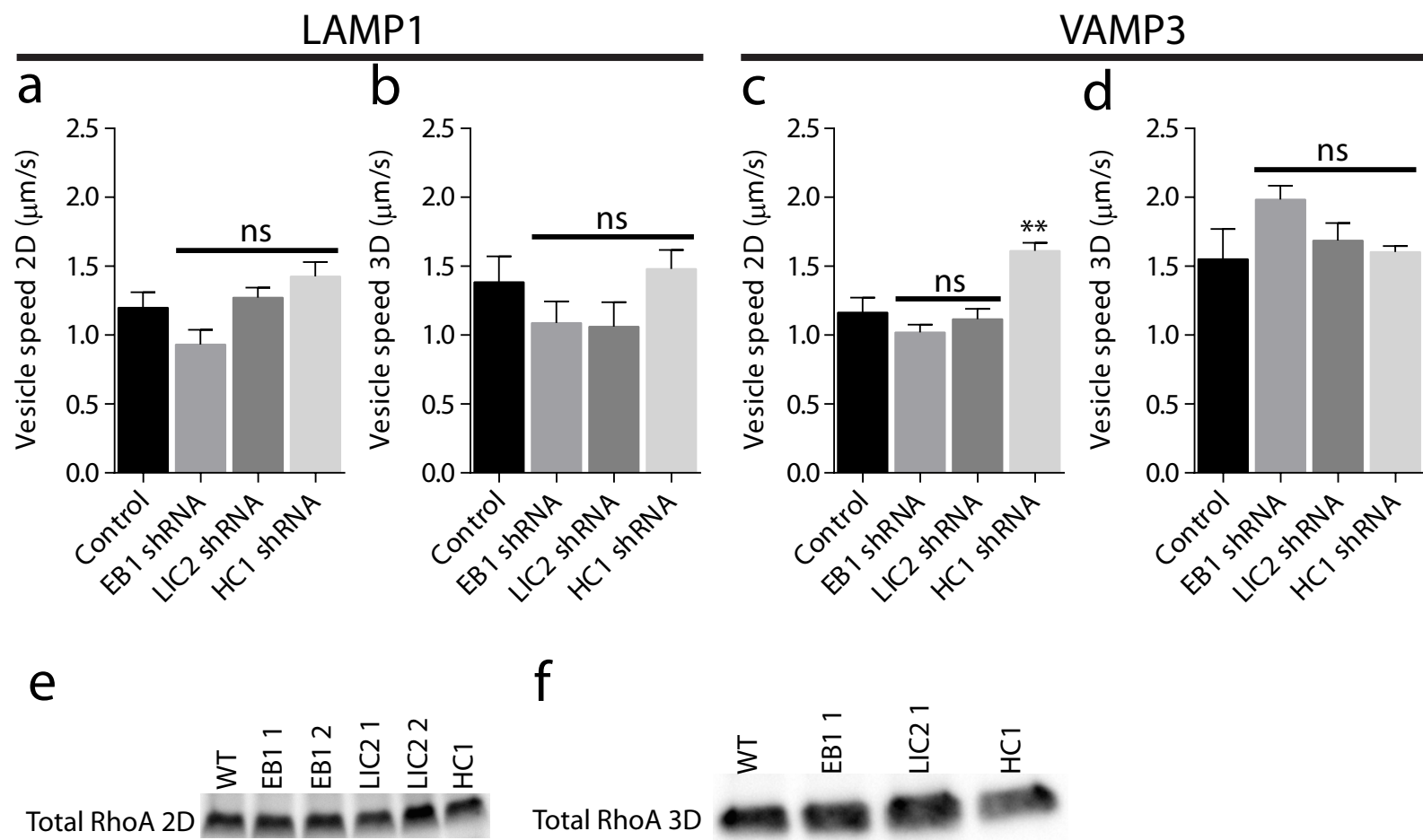

Supplemental figure 3 - Jayatilaka et al.

# Nocodazole

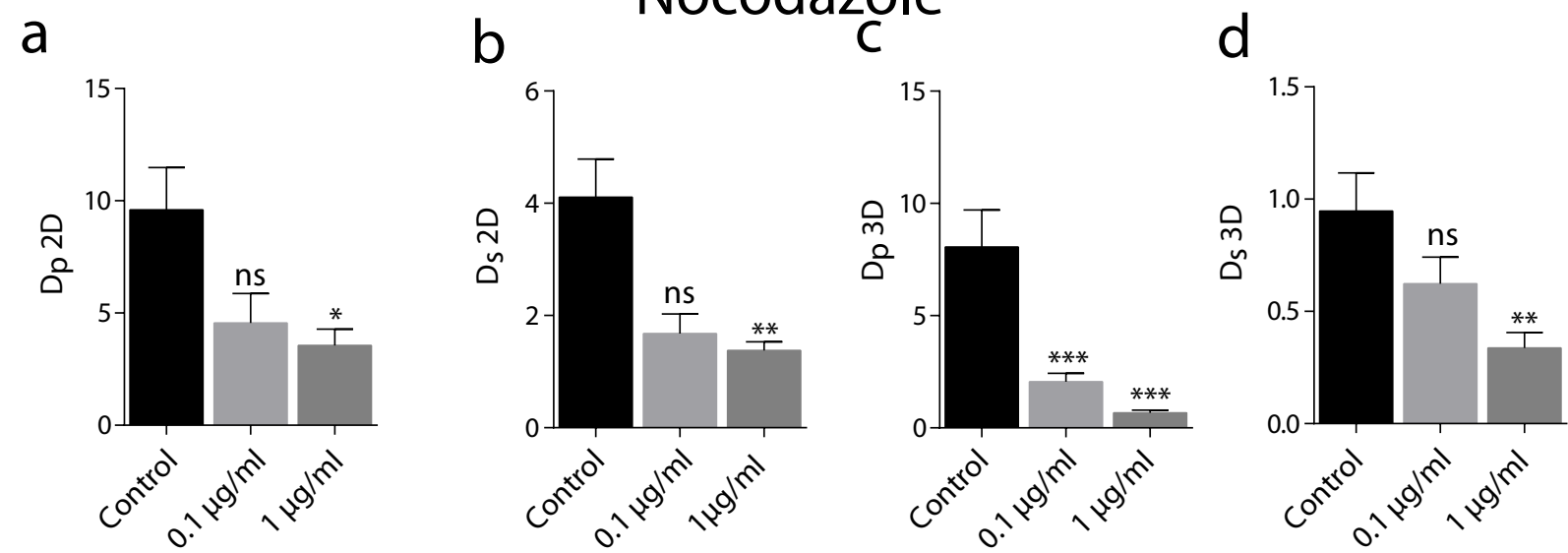

# Taxol

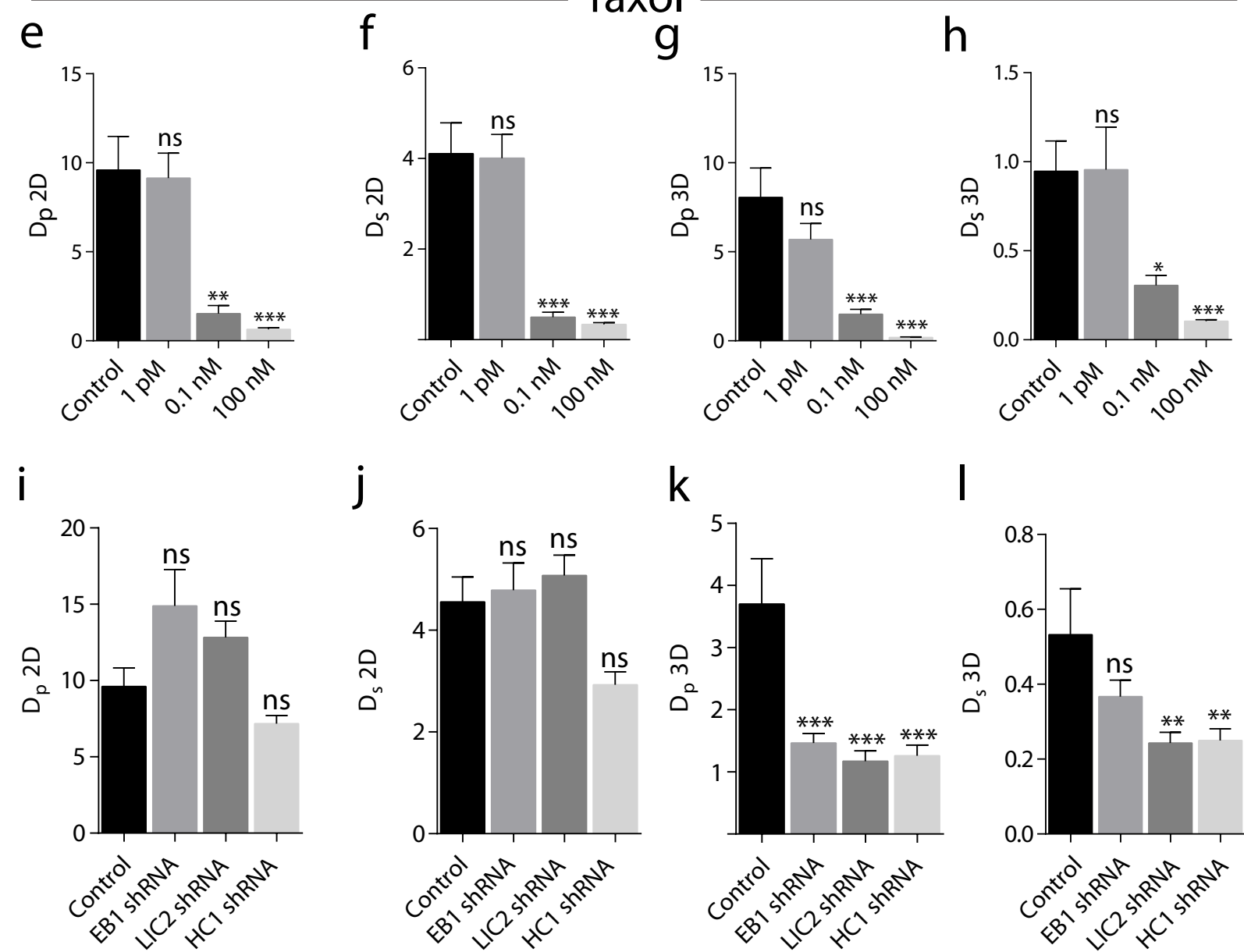

Supplement: Supplementary file 2 [file fj.201700444RR.sf1.pdf]
